# Sex-Specific sRNA Signatures in Rat Liver Reveal Divergent Alterations Following Perinatal Exposure to Glyphosate and Its Mixture with 2,4-D and Dicamba

**DOI:** 10.3390/ijms27104221

**Published:** 2026-05-09

**Authors:** Paraskevi Maria Nechalioti, Maria-Anna Kyrgiafini, Periklis Vardakas, Zoi Skaperda, Fotios Tekos, Charitini Nepka, Robin Mesnage, Michael N. Antoniou, Anca Oana Docea, Zissis Mamuris, Demetrios Kouretas

**Affiliations:** 1 Laboratory of Animal Physiology, Department of Biochemistry & Biotechnology, University of Thessaly, 41500 Larissa, Greece; pnechalioti@uth.gr (P.M.N.); pevardakas@uth.gr (P.V.); zoskaper@uth.gr (Z.S.); cnepka@yahoo.gr (C.N.); 2 Laboratory of Genetics, Comparative and Evolutionary Biology, Department of Biochemistry & Biotechnology, University of Thessaly, 41500 Larissa, Greece; mkyrgiafini@uth.gr (M.-A.K.); zmamur@bio.uth.gr (Z.M.); 3 Gene Expression and Therapy Group, Faculty of Life Sciences and Medicine, Department of Medical and Molecular Genetics, King’s College London, Guy’s Hospital, London SE1 9RT, UK; robin.mesnage@kcl.ac.uk (R.M.); michael.antoniou@kcl.ac.uk (M.N.A.); 4 Department of Toxicology, University of Medicine and Pharmacy of Craiova, 200349 Craiova, Romania

**Keywords:** toxicogenomics, noncoding RNAs, microRNA, herbicide mixtures, glyphosate, 2,4-D, dicamba, perinatal exposure

## Abstract

Perinatal exposure to environmental toxicants, even at regulatory relevant doses, can disrupt molecular programming during critical developmental windows, with long-term consequences for organ function and disease risk. We investigated sex-specific hepatic microRNA (miRNA) responses in Wistar rats following perinatal exposure to glyphosate at European Union (EU) acceptable daily intake (ADI) dose (0.5 mg/kg bw/day), at no-observed-adverse-effect level (NOAEL; 50 mg/kg bw/day), and mixed with 2,4-D (0.3 mg/kg bw/day) and dicamba (0.02 mg/kg bw/day), each at their ADI. Using small RNA sequencing, we identified distinct miRNA expression profiles in males and females, with the mixture inducing the most pronounced divergence (52 differentially expressed miRNAs between males and females). Functional enrichment analysis of validated miRNA targets revealed activation of apoptotic, oncogenic, and stress-related pathways in males, alongside downregulation of homeostatic and anti-fibrotic regulators. Females showed suppression of miRNAs involved in hormone signaling, development, and tissue regeneration, suggesting endocrine and adaptive disruption. Our findings highlight the importance of determining effects based on sex and sensitive developmental stages in toxicological assessment, since different regulatory programs may be involved in the response of males and females to xenobiotics. The identified miRNAs may represent early biomarkers of hepatic dysfunction following early-life herbicide exposure, supporting their utility in future risk evaluations.

## 1. Introduction

Glyphosate, an N-phosphonomethyl derivative of glycine, is the declared active compound in the most widely applied herbicide formulations globally [1]. Glyphosate exerts its herbicidal action by inhibiting 5-enolpyruvylshikimate-3-phosphate synthase (EPSPS) of the shikimate pathway, thereby blocking production of essential aromatic amino acids [2]. Given that the shikimate pathway is absent in animal species, including humans, glyphosate-based herbicides (GBHs) have been considered to have a very high safety profile. However, research over the years has demonstrated that glyphosate is associated with the occurrence of several adverse health outcomes, including inflammatory diseases [3], neurological disorders [4], reproductive disruptions [5], endocrine-disrupting properties [6], fatty liver disease [7] and carcinogenicity [8,9,10,11], with the International Agency for Research on Cancer (IARC) rating glyphosate and GBHs as Class 2A probable human carcinogens [12]. Underpinning these disease conditions are observations that glyphosate and GBHs induce oxidative stress [13], leading to genotoxicity [14,15], disruption of antioxidant defense systems [16,17], and interference with cellular signaling pathways [16,18].

Glyphosate is also frequently used in combination with other herbicides, such as 2,4-dichlorophenoxyacetic acid (2,4-D) and dicamba, for enhancing the effectiveness of weed control. 2,4-D and dicamba are the most commonly applied synthetic plant growth regulators, acting as selective herbicides against broadleaf weeds [19]. However, detection of 2,4-D in surface and groundwater is a common phenomenon, raising serious concern about its potential toxic effects in non-target organisms [20]. There is strong evidence linking 2,4-D exposure to the induction of oxidative stress, and the IARC has classified 2,4-D as a Class 2B possible human carcinogen [21]. There are likewise concerns about dicamba’s potential implications in human health, as herbicide exposure has been associated with an increased risk of developing liver and intrahepatic bile duct cancer [22].

In addition, human exposure to multiple pesticides, including herbicides, via multiple routes (e.g., diet, water, inhalation, and dermal absorption) is inevitable. Even though exposure occurs at regulatory permitted levels, the combination of two or more herbicides may result in synergistic, additive, or antagonistic effects, which can significantly modify their toxicological profile when compared with each compound alone. Notably, a major knowledge gap exists regarding cumulative effects of glyphosate and other herbicides on human health in realistic exposure scenarios [23,24]. The Real-Life Risk Simulation (RLRS) approach aims to address the need for a more comprehensive and accurate prediction of the biological response against chemical exposure. Therefore, RLRS evaluates low-dose, long-term, multi-chemical interactions, mimicking real-human exposure [24]. An RLRS approach can prove even more useful when considering exposures during critical developmental stages, such as pregnancy and the perinatal period, in which organs are still maturing and epigenetic programming is particularly sensitive to environmental stimuli [25,26]. Glyphosate exposure during pregnancy and early postnatal life may give rise to more pronounced toxic outcomes by disrupting developmental signaling pathways and ultimately causing serious molecular alterations [27]. In this regard, glyphosate exposure in a mouse model system has been found to induce large-scale epigenetic (DNA methylation) changes, leading to transgenerationally inherited negative health outcomes including prostate disease, obesity, kidney disease, ovarian disease, and parturition (birth) abnormalities [28,29]. Furthermore, alongside increasing use of GBH application in glyphosate-tolerant, genetically modified cropping systems, a parallel rise in reproductive problems and birth defects has been recorded [5,30].

Among molecular regulators mediating responses to environmental toxicants are microRNAs (miRNAs) [31,32]. These small, non-coding RNA molecules are mostly post-transcriptional regulators of gene expression and act by binding to complementary sequences in target messenger RNAs (mRNAs), resulting in their degradation or translational repression [33]. MiRNAs play an essential role in fundamental biological processes, including embryonic development, tissue differentiation, metabolic regulation and cellular stress response [34]. Their expression is finely tuned and dynamically responsive to both internal and external stimuli [35]. Notably, exposure to xenobiotics, including environmental pollutants and pharmaceuticals, has been shown to alter miRNA expression profiles, disrupting critical signaling pathways and thus contributing to toxicological outcomes [31,32]. These characteristics make miRNAs not only central mediators of toxicity but also valuable early-stage biomarkers, providing insights into molecular mechanisms involved in damage before overt physiological symptoms appear [36]. In this context, profiling miRNA changes in target organs may provide a sensitive and mechanistically informative approach to understanding the biological impact of environmental chemicals.

Growing evidence suggests that miRNAs are expressed in a sex-dependent manner, with distinct expression levels and patterns observed in males and females across various tissues [37,38]. These differences are considered biologically significant, as miRNAs post-transcriptionally regulate gene expression and contribute to the establishment and maintenance of sex-specific traits, including those related to development, metabolism, and immune function [37]. Sex has long been recognized as a critical factor determining disease susceptibility, treatment response, and clinical outcomes, and recent studies have also highlighted its role in the occurrence of toxicological responses to environmental exposures [39]. Numerous chemical contaminants, including pesticides, endocrine-disrupting chemicals, and other environmental pollutants, have been shown to exert sex-specific effects, with males and females presenting divergent molecular and physiological responses [40,41,42]. Interestingly, sexually dimorphic metabolic effects have also been demonstrated after exposure to low doses of pesticide mixtures [43]. Several stressors may differentially modulate miRNA expression, resulting in sex-biased gene regulation [44] and potentially contributing to diverse risk of disease later in life. Despite these observations, current studies examining sex-specific miRNA responses to environmental toxicants are limited in number and scope, underscoring the need for more research in this area.

The study presented here aimed to investigate how perinatal exposure to glyphosate alone and glyphosate combined with two other herbicide active ingredients, 2,4-D and dicamba, at regulatory relevant doses modulates sex-dependent differences in the small RNA (sRNA) expression landscape in rat liver, within and across exposure conditions. This mixture of herbicides was chosen as the use of 2,4-D and dicamba along with glyphosate has vastly increased in the last 10 years [45,46] due to the appearance of glyphosate-resistant weed species [47,48] and the launch of glyphosate-plus-dicamba- and glyphosate-plus-2,4-D-tolerant, genetically modified crops (soybeans, maize, cotton, etc.) [49], especially in North America, which in turn has resulted in heightened human exposure [50]. Using high-throughput small RNA sequencing (sRNA-seq), we profiled liver samples from male and female Wistar rat offspring that were exposed to these compounds starting at a prenatal stage of development until 13 weeks post-weaning, simulating environmentally relevant exposure conditions. The objectives were to identify differentially expressed miRNAs and to provide a preliminary assessment of sex-dependent divergence in hepatic miRNA expression across exposure conditions. Through this analysis, we aimed to provide insights into the role of miRNAs in shaping sex-dependent regulatory responses under baseline and exposure-modulated conditions, contributing to a better understanding of the molecular complexity of mixture toxicology and developmental exposure.

## 2. Results

### 2.1. Small RNA Sequencing and Data Quality

Small RNA-seq was conducted on 16 sequencing pools (two biological replicates per experimental group) of liver samples from male and female Wistar rat offspring from control and herbicide-exposed groups. Exposure was commenced at gestational day 6 and continued to 13 weeks post-weaning. The exposure groups were glyphosate alone at EU ADI (0.5 mg/kg bw/day) and NOAEL (50 mg/kg bw/day) doses and a glyphosate, 2,4-D (0.3 mg/kg bw/day), and dicamba (0.02 mg/kg bw/day) mixture, with each at the EU ADI dose. This study was designed to investigate sex-specific alterations in miRNA expression profiles, with a particular focus on the identification of differentially expressed (DE) miRNAs between male and female rats in response to herbicide exposure commencing at an early stage of life.

The quality of small RNA-seq data for each liver sample is summarized in Table S1. The data are of high quality and consistent across all analyzed samples. The total read count averaged 10,208,141, with individual sample values ranging from 8,612,105 in female control group 1 (FC1) to 11,660,410 in female herbicide mixture group 1 (FMix1). After stringent quality control filtering, the average clean read count was 9,774,780, with values ranging from 8,009,440 (FC1) to 11,356,894 in male herbicide mixture group 2 (MMix2). Retention rates of clean reads were between 91.92% and 99.4% of total reads. The mapping efficiency of clean reads to the reference genome was similarly robust, with an average of 95.54% across all samples. Overall, the sequencing performance was consistent across experimental groups, providing a strong foundation for downstream analyses, including miRNA identification, differential expression, and functional enrichment.

### 2.2. Identification of Known and Novel miRNAs

Small RNA-seq analysis revealed a total of 422 known mature miRNAs and 50 novel mature miRNAs across all liver samples (Figure S1). In female rats, the number of mapped mature miRNAs per sample ranged from 245 in the FC1 sample to 302 in the female herbicide mixture 1 sample (FMix1), while the number of corresponding hairpin miRNAs ranged from 234 (FC1) to 281 (FMix1). Importantly, no statistically significant differences were observed in the number of known mature or hairpin miRNAs among the biological replicates, indicating consistency across samples within treatment groups (Figure S1).

In male rats, the number of mapped mature miRNAs was slightly higher than in the female samples, ranging from 249 in male control group 2 (MC2) sample to 326 in male herbicide mixture group 1 (MMix1) (Figure S2). Similarly, the number of mapped hairpin miRNAs ranged from 238 (MC2) to 299 (MMix1). As observed in the female cohort, no statistically significant differences were detected between biological replicates in either mature or hairpin miRNA counts, indicating reliable detection across samples (Figure S2).

The number of novel mature miRNAs in female rats ranged from 18 in control group 2 (FC2) to 30 in herbicide mixture group 1 (FMix1), while the number of novel hairpin miRNAs varied from 21 (FC1, FC2) to 34 in herbicide mixture group 2 (FMix2) (Figure S3). Although no statistically significant differences were observed between replicates, an increase in the number of novel miRNAs was noted across the treatment groups compared with the controls, suggesting a potential exposure-related effect on miRNA biogenesis or detection (Figure S3).

In male rats, the number of novel mature miRNAs ranged from 19 in control group 2 (MC2) to 37 in herbicide mixture group 1 (MMix1), while the number of novel hairpin miRNAs varied from 21 in control groups 1 and 2 (MC1 and MC2) to 39 in MM1 (Figure S4). Although no statistically significant differences were observed between biological replicates, a marked increase in the number of novel miRNAs was detected in the herbicide mixture-exposed group (MMix). Notably, the number of novel mature and hairpin miRNAs in the MM group was approximately double that of the control group, suggesting a potential effect of the herbicide mixture on miRNA biogenesis or expression (Figure S4).

### 2.3. Identification of Differentially Expressed (DE) miRNAs

**Figure 1:**
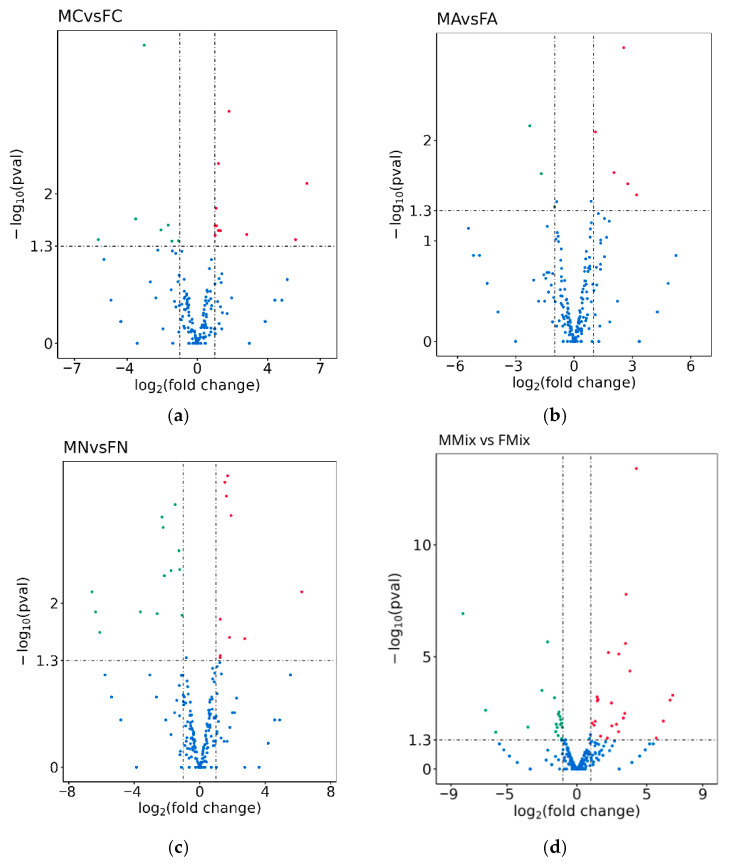
Volcano plots illustrating differentially expressed miRNAs between male and female rats. Significant miRNAs were identified based on adjusted *p*-values (<0.05) and |log_2_ (fold change)| > 1. Upregulated miRNAs are shown in red, and downregulated miRNAs are shown in green. Non-significantly differentially expressed miRNAs are shown in blue. Panels show the following comparisons: (**a**) Male control vs. female control (MC vs. FC) groups. (**b**) Male ADI vs. female ADI (MA vs. FA) groups. (**c**) Male NOAEL vs. female NOAEL (MN vs. FN) groups. (**d**) Male herbicide mixture vs. female herbicide mixture (MMix vs. FMix) groups.

To investigate sex-specific responses, we compared miRNA expression between female and male rats within each treatment group (FA vs. MA, FN vs. MN, and FMix vs. MMix), as well as between control males and females (MC vs. FC) (Figure 1). In the control comparison, 14 miRNAs were upregulated and 9 were downregulated in males (MC vs. FC) (Figure 1a). The glyphosate ADI group exhibited the fewest sex-related differences, with five miRNAs being upregulated and three being downregulated in males compared with females (MA vs. FA) (Figure 1b). In contrast, the glyphosate NOAEL group (MN vs. FN) showed a more pronounced sex bias, with eleven miRNAs being upregulated and thirteen being downregulated in male rats compared with females (Figure 1c). Herbicide mixture ADI exposure resulted in the largest sex-specific divergence, with thirty-one miRNAs being upregulated and twenty-one being downregulated in male rats compared with female rats (MM vs. FMix) (Figure 1d). The full list of differentially expressed miRNAs is provided in Table S2.

To further characterize sex-specific differences, the top differentially expressed miRNAs for each comparison are summarized in Table 1. In the control group (MC vs. FC), a balanced pattern of up- and downregulated miRNAs was observed, indicating baseline sex-dependent differences in hepatic miRNA expression. These differences were also reflected in substantial variation in expression magnitude between sexes under control conditions. In the glyphosate ADI group (MA vs. FA), fewer miRNAs were differentially expressed, and the observed fold changes were generally of lower magnitude, suggesting a relatively limited impact of this exposure level on sex-specific regulation. In contrast, the NOAEL group (MN vs. FN) exhibited more pronounced divergence, characterized by both an increased number of deregulated miRNAs and larger fold changes, indicating enhanced sex-dependent regulatory differences at higher exposure levels. Notably, the herbicide mixture group (MMix vs. FMix) showed the strongest sex-specific response, with the largest magnitude changes observed across all comparisons. Among the most markedly altered miRNAs, rno-miR-19b-3p and rno-miR-224-5p exemplify the extent of this divergence, indicating that combined exposure amplifies intrinsic sex-dependent differences in hepatic miRNA regulation.

In addition to known miRNAs, as shown in Table S2, novel candidates were also detected. Specifically, two novel miRNAs were found to be downregulated in males compared with females in the glyphosate NOAEL group (MN vs. FN), while in the herbicide mixture ADI group (MMix vs. FMix), five novel miRNAs were upregulated, and one was downregulated.

Overall, these results point out the presence of sex-specific differences in hepatic miRNA expression. The relatively small number of DE miRNAs at the ADI level suggests a limited impact of low-dose exposure. In contrast, the stronger divergence observed at the glyphosate NOAEL, and more prominently in the mixture ADI group, highlights that both dose and combined exposure amplify sex-dependent miRNA responses.

### 2.4. Functional Enrichment Analysis of DE miRNAs

To gain insight into the biological consequences of the observed differences in miRNA profiles between male and female rats, we conducted a comprehensive target gene analysis of the DE miRNAs. To identify the biological processes, cellular components, and molecular functions associated with miRNA target genes, we performed Gene Ontology (GO) [51,52] and Kyoto Encyclopedia of Genes and Genomes (KEGG) pathway [53] enrichment analyses. These analyses allowed us to identify key molecular pathways and cellular mechanisms that are potentially affected by the observed sex-specific miRNA differences in response to herbicide exposure.

**Figure 2:**
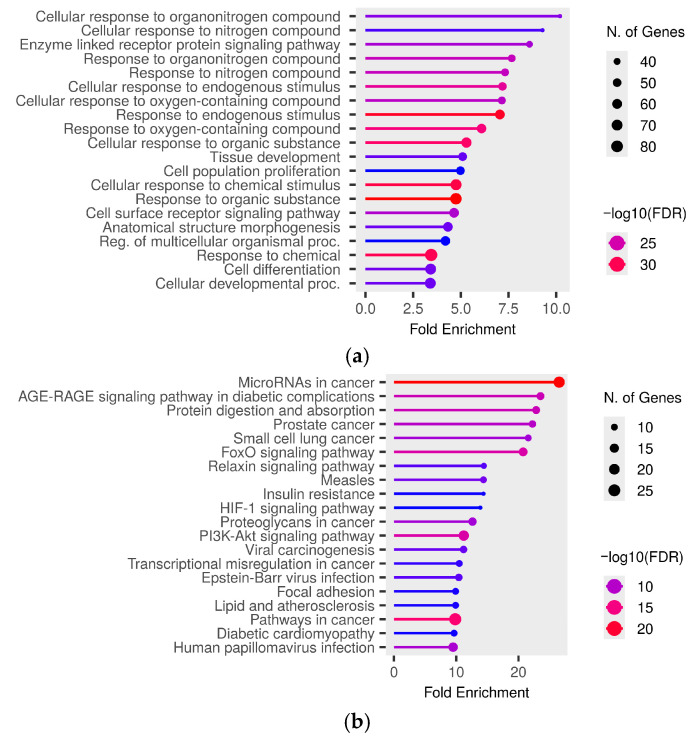
Significant (**a**) GO Biological Process and (**b**) KEGG pathway terms associated with the overlap gene targets of DE miRNAs. The size and color of the dots represent the number of genes and the range of statistical significance, respectively. The red color indicates higher −log10(FDR) values, followed by pink, purple and blue colors. The y-axis represents the GO and KEGG terms, and the x-axis represents fold enrichment. The *p*-values were corrected for multiple tests using the false discovery rate (FDR).

According to miRTargetLink 2.0 [54], we identified the overlap of validated target genes among all DE miRNAs (Table S3). Subsequently, GO and KEGG enrichment analyses were performed. As shown in Figure 2a, the most significantly enriched GO Biological Process terms included “cellular response to nitrogen compound”, “response to organonitrogen compound”, “response to organic substance”, “response to chemical stimulus”, “cell population proliferation”, and “cell differentiation”. For KEGG pathways, the top-enriched term was “microRNAs in cancer”. Other enriched terms included “AGE–RAGE signaling pathway in diabetic complications”, “protein digestion and absorption”, “FoxO signaling pathway”, “insulin resistance”, and “PI3K–Akt signaling pathway” (Figure 2b). The results for the GO Cellular Component and Molecular Function categories are provided in Figure S5 and Figure S6, respectively.

Subsequently, we focused on the comparison that exhibited the greatest number of DE miRNAs, MMix vs. FMix, representing male and female rats exposed to the herbicide mixture at the ADI. For this comparison, we identified the overlap of validated target genes for both upregulated (Table S4) and downregulated miRNAs (Table S5) using miRTargetLink 2.0 [54], followed by GO and KEGG enrichment analyses.

**Figure 3:**
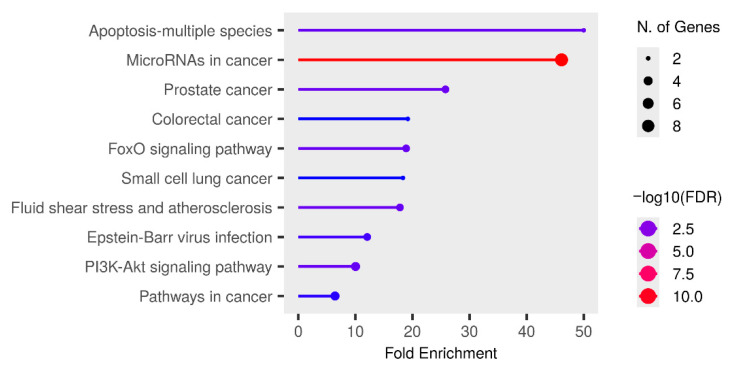
Significant KEGG pathway terms associated with the overlap of gene targets of upregulated miRNAs in the male herbicide mixture vs. female herbicide mixture (MMix vs. FMix) rat groups. The size and color of the dots represent the number of genes and the range of statistical significance, respectively. The red color indicates higher −log10(FDR) values, followed by pink, purple and blue colors. The y-axis represents the KEGG terms, and the x-axis represents fold enrichment. The *p*-values were corrected for multiple tests using the false discovery rate (FDR).

For miRNAs upregulated in males, the most enriched KEGG pathways included “apoptosis”, “microRNAs in cancer”, “FoxO signaling pathway”, and “PI3K–Akt signaling pathway”, along with several pathways associated with various cancer types (Figure 3). The results for the GO Molecular Function and GO Biological Process categories are presented in Figure S7 and Figure S8, respectively. No statistically significant enrichment was observed for the GO Cellular Component category.

**Figure 4:**
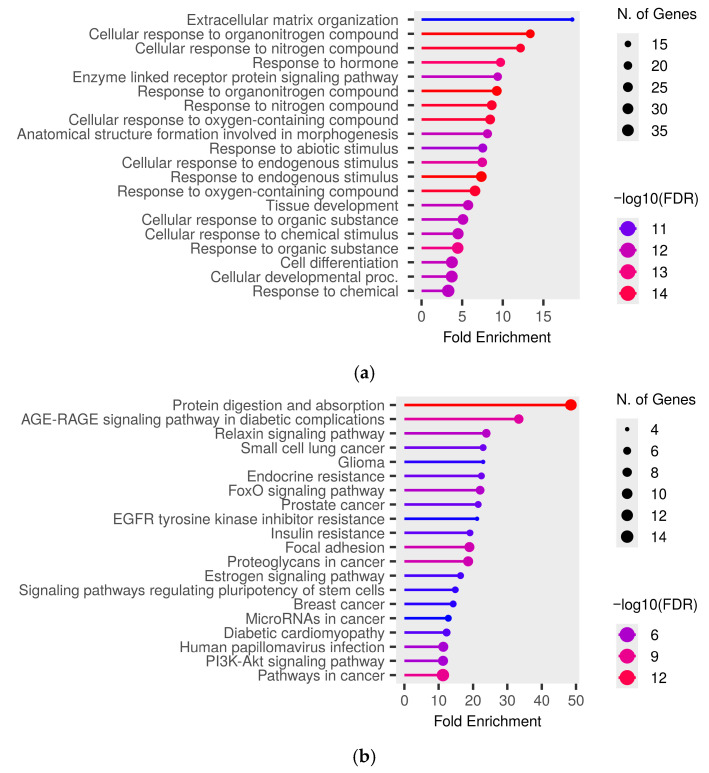
Significant (**a**) GO Biological Process and (**b**) KEGG pathway terms associated with the overlap of gene targets of downregulated miRNAs in the male herbicide mixture vs. female herbicide mixture (MMix vs. FMix) rat groups. The size and color of the dots represent the number of genes and the range of statistical significance, respectively. The red color indicates higher −log10(FDR) values, followed by pink, purple and blue colors. The y-axis represents the GO and KEGG terms, and the x-axis represents fold enrichment. The *p*-values were corrected for multiple tests using the false discovery rate (FDR).

For miRNAs downregulated in males, the most enriched GO Biological Process terms were “extracellular matrix organization”, “cellular response to organonitrogen compound”, “cellular response to nitrogen compound”, “cellular response to organic substance”, “tissue development”, and “cell differentiation” (Figure 4a). Similarly, the top-enriched KEGG term was “protein digestion and absorption” (Figure 4b). The results for the GO Cellular Component and Molecular Function categories are provided in Figure S9 and Figure S10, respectively.

Taken together, our findings suggest that exposure to the glyphosate, 2,4-D and dicamba mixture at EU ADI elicits sex-dependent molecular responses in the liver. Such differences may reflect distinct mechanisms of hepatic adaptation between sexes under exposure to this herbicide mixture.

## 3. Discussion

The present investigation constitutes the first to explore potential sex-specific hepatic miRNA alterations in response to perinatal exposure to glyphosate alone and its combination with 2,4-D and dicamba at regulatory relevant doses using small RNA sequencing in male and female Wistar rats. The liver was chosen for his investigation since it is the principal site of xenobiotic metabolism and body detoxification, with this organ being particularly vulnerable to herbicide insult. Previous studies have shown that GBHs can exert hepatotoxic effects and cause hepatic alterations in rat model systems and humans, even at regulatory permissible doses [55,56]. In particular, some early studies suggest that perinatal exposure may affect long-term liver physiology [57,58,59]. However, the available data are scarce, and further investigation is required.

Sex differences in liver biology are well-documented and influence various physiological and pathological processes, including xenobiotic metabolism, hormone signaling, and susceptibility to liver disease [60,61,62,63]. The miRNA system plays a crucial regulatory role in maintaining hepatic homeostasis and physiology [64,65] and has been implicated in the organism’s response to toxicants and environmental stressors [31,32].

We identified baseline differences in miRNA expression between the two sexes, which were further modified by exposure to the herbicide active ingredients. Among all exposure groups, the ADI glyphosate, 2,4-D, dicamba mixture was linked to the most pronounced sex-dependent divergence in miRNA profiles (52 DE miRNAs). Functional analysis of validated target genes revealed that DE miRNAs between males and females were associated with distinct biological processes and signaling pathways. These findings might suggest that exposure elicited sex-dependent molecular responses in the liver under exposure conditions, potentially reflecting differences in susceptibility, stress adaptation, or detoxification capacity between males and females.

Sex-based differences in hepatic miRNA alterations observed in this study are consistent with known sexual dimorphism in liver function, xenobiotic metabolism, and pathophysiology [60,61,62,63]. Hepatic sexual dimorphism refers to the sex-specific differences presented in liver structure, function, and gene expression. Gene expression analysis in murine livers has identified up to 6000 differentially expressed genes between sexes, revealing significant variations in liver physiology, metabolism, and disease susceptibility [66]. The primary factors driving hepatic sexual dimorphism include sex hormones, developmental factors, growth hormone (GH) secretion, and morphogens [63,66]. Furthermore, metabolites produced by the gut microbiota affect male-specific GH patterns, further enhancing these differences [66]. Such disparities can lead to divergent outcomes following toxicant exposure, as demonstrated in previous, albeit limited, studies [67,68]. The role of miRNAs in hepatic sexual dimorphism, particularly within a toxicological context, is also underexplored.

In the present study, perinatal exposure to glyphosate and a glyphosate mixture with 2,4-D and dicamba appears to be linked to sex-specific alterations in hepatic miRNA expression. Males exhibited a distinct molecular signature characterized by the upregulation of oncogenic and apoptotic miRNAs, along with the downregulation of homeostatic and protective regulators. Among the upregulated miRNAs in males, several are closely linked to tumorigenesis and cellular stress. The findings we present here are in accord with previous research, which revealed alterations in miRNAs involved in carcinogenesis in female rats administered with EU NOAEL and ADI doses of glyphosate alone and its formulated product MON 52276 [69]. Similar changes in liver gene expression and miRNA profiles were also observed in female rats exposed to an EU ADI pesticide mixture including glyphosate [70].

MiR-21-5p (upregulated in MC vs. FC and MA vs. FA exposure groups), is a well-established oncomiR, significantly upregulated in liver cancer and supporting tumor growth and progression [71]. Similarly, miR-224-5p (upregulated in the MMix vs. FMix group comparison) has been implicated in multiple cancer types, promoting invasion, migration, and proliferation of cancer cells while inhibiting apoptosis [72,73,74]. Another miRNA consistently upregulated across three comparisons (MC vs. FC, MN vs. FN, and MMix vs. FMix) is miR-125b, a pleiotropic regulator involved in tumor cell proliferation, differentiation, invasion, migration, drug resistance and tumor immunity [75]. Notably, recent studies also indicate that miR-125b plays a sex-dependent regulatory role in liver fibrosis, particularly through its interaction with androgen receptor (AR) signaling, TGF-β, and apelin pathways, thereby influencing male-biased hepatic vulnerability [76]. Furthermore, miR-183-5p (upregulated in MN vs. FN and MMix vs. FMix) has been associated with cancer progression [77]. Collectively, the upregulation of specific miRNAs might suggest a coordinated activation of pro-apoptotic, oncogenic, and metabolic stress pathways in male livers, potentially increasing susceptibility to hepatotoxic injury and long-term carcinogenic risk under glyphosate-based mixture exposure.

In parallel, several miRNAs downregulated in males are known to play key roles in maintaining liver homeostasis and suppressing pathological remodeling. One of the most consistently downregulated miRNAs across exposure group comparisons (MC vs. FC, MN vs. FN, and MMix vs. FMix) was miR-29b-3p, a well-characterized regulator of extracellular matrix (ECM) turnover and anti-fibrotic signaling [78]. Specifically, members of the miR-29a/b family have been related to several protective effects in renal disease, particularly with regard to fibrosis, inflammation, apoptosis, and kidney injury [79,80]. Research also indicates that miR-29b plays a hepatoprotective role against alcohol-induced inflammation and liver injury by targeting STAT3 [81]. Another downregulated miRNA, miR-203a-5p (MMix vs. FMix groups), has been shown to confer protective effects against cytokine-induced tissue injury by modulating suppressor of cytokine signaling (SOCS) proteins and related inflammatory cascades [82]. Although most evidence stems from renal models, SOCS family members also play a central role in liver inflammation and regeneration, suggesting that reduced miR-203a-5p expression may facilitate dysregulated immune signaling and impair the liver’s adaptive response to injury. Taken together, the downregulation of fibrosis-limiting and inflammation-modulating miRNAs in male rat liver might suggest a loss of regulatory balance that may predispose to fibrogenesis, reduced tissue repair capacity, and chronic damage due to glyphosate, 2,4-D, dicamba herbicide mixture exposure.

Although the most prominent miRNA deregulation was observed in male rats, both sexes exhibited molecular alterations following herbicide exposure. However, the underlying mechanisms appear to diverge. While males displayed activation of oncogenic and stress-related miRNAs, the female response was characterized by suppression of regenerative and endocrine-regulated processes, rather than overt tumor-promoting activity. In contrast to males, females showed a relative upregulation of several miRNAs associated with liver homeostasis and stress resilience, including miR-29b-3p and miR-203a-5p, which may have a protective role. Females also exhibited shifts in miRNA expression suggestive of endocrine and developmental disruption. For example, miR-7a-5p, which is downregulated in the MMix vs. FMix group comparison, may regulate pituitary development and reproduction. A recent study indicates that miR-7a-5p overexpression attenuates FSHb expression and decreases FSH secretion [83]. Another downregulated miRNA was miR-143 in females in the MMix vs. FMix group comparison. This miRNA is highly enriched in the mouse ovary, regulating hormone production and other cellular processes, and plays a role in steroidogenesis, including the production of estradiol, progesterone, and testosterone, by targeting genes involved in these pathways [84]. Therefore, their reduced expression in female liver may reflect interference with hormone-dependent regulatory networks. Additionally, several miRNAs that were downregulated in females might suggest interference with developmental and regenerative programs, possibly through endocrine-modulated pathways. This pattern implies that exposure to the glyphosate, 2,4-D, and dicamba herbicide mixture may compromise the adaptive and repair capacity of female livers through more subtle hormonal and developmental interference.

These miRNA patterns align functionally with our enrichment analyses: male-biased miRNAs targeted pathways related to apoptosis, AGE–RAGE signaling, and microRNAs in cancer, whereas female-enriched miRNAs were associated with extracellular matrix organization, hormone-regulated signaling and tissue development. This discrepancy may indicate a more protective regulatory environment in females that is less disrupted by herbicide mixture exposure, whereas males appear more vulnerable to oncogenic and metabolic disturbances. Such sex-specific molecular responses are biologically plausible and are in line with the established sexual dimorphism in liver, which is governed by lifelong effects of sex hormones, growth hormone (GH) secretion patterns and developmental programming [60,63,66]. In males, pulsatile GH secretion activates the STAT5b transcription factor, promoting the male-specific expression of detoxification, metabolic and inflammatory genes. Contrariwise, the more continuous GH pattern in females supports distinct transcriptional programs linked to regeneration and endocrine function [85,86]. Several key miRNAs modified in this investigation may interact with these sex-specific axes. For example, miR-125b, upregulated in males, has been implicated in liver fibrosis in a sex-dependent manner through androgen receptor (AR) signaling, TGF-β, and apelin pathways [76]. The male-specific downregulation of miR-29b-3p, a potent anti-fibrotic and anti-inflammatory miRNA [78,87], might further support the notion of disrupted tissue maintenance and repair mechanisms in males compared with females. On the contrary, several miRNAs downregulated in mixture-treated females, like miR-7a-5p and miR-143, are known regulators of pituitary function, steroidogenesis, and reproductive signaling, probably suggesting that endocrine-sensitive pathways are key targets in female livers [83,84]. Their suppression may indicate mixture-induced disturbances in hormone-dependent regenerative programs, potentially compromising adaptability without triggering overt carcinogenic signaling. Collectively, these results might suggest that sex hormones and GH-regulated signaling may underlie the distinct molecular trajectories observed within the glyphosate, 2,4-D, and dicamba mixture group, rendering males and females potentially more vulnerable to pro-oncogenic responses and compromised homeostatic and hormonal adaptability, respectively.

Importantly, the interpretation of the present miRNA findings should be considered within the broader biological context of the experimental model. In the same set of animals, liver biochemical and histopathological changes have been demonstrated in both sexes, with more pronounced effects in the mixture group [59]. While these findings do not constitute direct functional validation of the miRNA-based inferences, they might support the biological relevance of the hepatic responses identified in the present study. In this context, the observed sex-dependent divergence in miRNA expression may reflect underlying regulatory mechanisms associated with these phenotypic differences.

**Figure 5:**
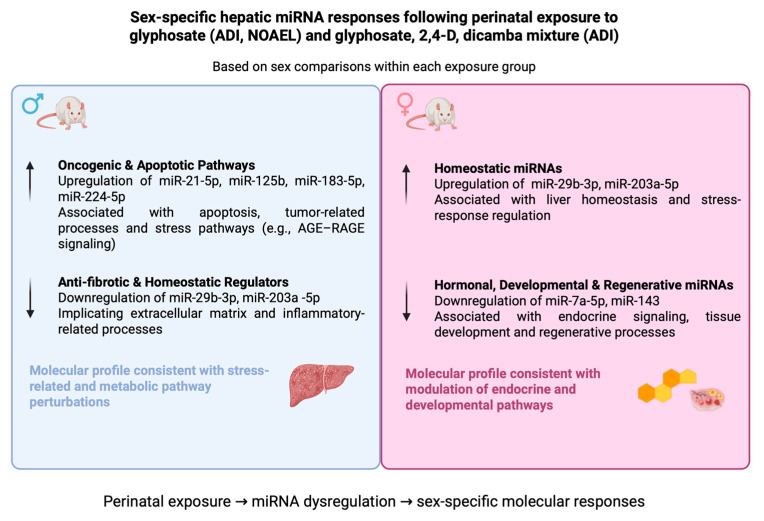
Sex-specific hepatic miRNA regulatory patterns following perinatal exposure to glyphosate and a glyphosate, 2,4-D, and dicamba mixture. Upward arrows indicate upregulation, whereas downward arrows indicate downregulation. Blue and pink panels represent male- and female-associated responses, respectively. Created in BioRender. nechalioti, M. (2026) https://BioRender.com/7ydp9km.

The detected sex-specific miRNA expression profiles indicate that male and female rats respond in a divergent manner to the herbicides studied here, leading to distinct vulnerabilities. In males, the upregulation of miRNAs related to oncogenic and apoptotic pathways, along with the downregulation of anti-fibrotic and homeostatic regulators, might suggest increased susceptibility to hepatocellular injury, fibrogenesis, and metabolic dysregulation. In contrast, females exhibited suppression of miRNAs that regulate hormonal signaling, tissue development and regenerative processes. This pattern might reflect a more subtle form of disruption that may compromise endocrine balance and limit hepatic adaptability (Figure 5).

Considering that the perinatal period is critical to epigenetic and transcriptional programming of liver function [88,89], and that the liver undergoes extensive maturation during this developmental window, including the establishment of sex-specific gene expression patterns and hormonal responsiveness [90], disruption of miRNA regulatory networks at this juncture may influence pathways related to metabolism, tissue repair and endocrine function. Such miRNA alterations in early life may “prime” the liver for increased susceptibility to chronic diseases later in life, including fibrosis, metabolic dysfunction and hormone-related disorders. Notably, several of the deregulated miRNAs identified in the present study, such as miR-125b, miR-29b-3p, and miR-143 have established roles in liver pathology, thus strengthening their potential as early biomarkers of chemically induced hepatic dysfunction.

A key strength of our investigation research is the prenatal commencement of its focus on early-life exposure, which encompasses a critical developmental window that is often underrepresented in toxicogenomic studies, particularly with regard to sex-based molecular responses [88,89,90]. Furthermore, the evaluation of glyphosate alone and its combination with 2,4-D and dicamba reflects more accurately the real-world exposure scenarios conditions to mixtures of xenobiotics. Moreover, the integration of differential expression data with pathway enrichment enabled the identification of biologically relevant regulatory patterns.

However, it is important to acknowledge certain limitations of the study, such as the lack of experimental validation of the predicted miRNA–mRNA interactions. While small RNA sequencing provides comprehensive miRNA profiling, the functional validation of key targets (e.g., via qPCR or other molecular assays) was beyond the scope of the current work. The expression levels of key differentially expressed miRNAs were not validated using RT-qPCR in individual, non-pooled samples, which would provide additional confidence in the observed patterns. Therefore, both the identified miRNAs and their predicted targets should be interpreted as putative, candidate signatures, based on sequencing data bioinformatic predictions, requiring further validation in future studies. Second, the relatively small effective sample size for small RNA sequencing, combined with the use of pooled samples, limits the assessment of inter-individual variability and reduces statistical power for differential expression and downstream analyses, providing a preliminary assessment of sex-dependent divergence in hepatic miRNA expression across exposure conditions. Moreover, novel miRNAs identified in this study should be interpreted as putative candidates, requiring confirmation through independent experimental validation. Finally, although offsprings were derived from multiple litters (five dams/per group), litter was not explicitly included in the statistical analysis, which does not allow for the complete exclusion of possible intra-litter correlation, underscoring the need for further confirmation. Future studies with larger cohorts and individual-level sequencing are needed to validate these results.

## 4. Materials and Methods

### 4.1. Study Design and Animal Experimentation

The experimental design has been described in detail in “Glyphomix Protocol” [91] and is based on Organization for Economic Co-operation and Development (OECD) Test Guideline (TG) 414. Wistar rats represent a well-established experimental model in toxicological studies with physiological and metabolic similarities to the human organism, including xenobiotic metabolism and liver function [92,93,94]. Briefly, three-month-old female Wistar rats (~300 g) were kept in sawdust-lined cages under standard housing conditions (12 h light/dark cycle, 19–23 °C, 35–55% humidity). Rats were given ad libitum access to a standard rodent diet and filtered water containing the test substances, as described below, where appropriate (Cantacuzino Institute, Bucharest, Romania). All animal procedures were conducted in accordance with Directive 2010/63/EU for the welfare of animals used for scientific purposes and were approved by the Ethics Committee of the University of Medicine and Pharmacy of Craiova, Romania (Approval No. 120/19.11.2020) and the National Sanitary Veterinary and Food Safety Authority (Approval No. 9/31.12.2020).

Test substances were administered via drinking water. Glyphosate (CAS No. 1071-83-6, Glyphosate PRESTANAL^®^; analytical standard; ≥98.0% purity), 2,4-D (2,4-dichlorophenoxyacetic acid; CAS No. 94-75-7, 97% purity) and dicamba (CAS No. 1918-00-9, Dicamba PRESTANAL^®^; analytical standard; ≥98.0% purity) were purchased from Sigma-Aldrich, Merck KGaA, Darmstadt, Germany. Glyphosate was given at the corresponding regulatory doses: the EU acceptable daily intake (ADI) of 0.5 mg/kg bw/day and the no-observed-adverse-effect level (NOAEL) of 50 mg/kg bw/day [95]. A herbicide mixture composed of glyphosate (0.5 mg/kg bw/day), 2,4-D (2,4-dichlorophenoxyacetic acid) (0.3 mg/kg bw/day) [96], and dicamba (0.02 mg/kg bw/day) [97] was also administered, with each compound provided at its respective EU ADI.

Twenty pregnant Wistar rats were randomly allocated into four groups (*n* = 5 dams per group): a control group receiving only standard diet and drinking water, two glyphosate groups receiving glyphosate at the EU acceptable daily intake (ADI) and the no-observed-adverse-effect level (NOAEL), and a fourth mixture group exposed to a mixture of glyphosate, dicamba, and 2,4-D at their respective EU ADI doses. Exposure to the tested substances began on gestational day (GD) 6 and continued through gestation and lactation until postnatal day (PND) 28. After weaning, the offspring were separated from the dams and maintained on the same exposure regimen for an additional 13 weeks, in accordance with OECD 90-day toxicological study requirements.

Each group of offspring consisted of ten males and ten females, with approximately two animals of each sex selected per litter. At the end of the 13-week exposure period, the animals were anesthetized with a mixture of xylazine (Alfazyne 2%, Alfasan Int., Woerden, The Netherlands) and ketamine (Alfamine 10%, Alfasan Int., Woerden, The Netherlands) and euthanized. Liver tissues were collected from the offspring, rinsed in ice-cold isotonic saline, snap-frozen in liquid nitrogen and maintained at −80 °C until further analysis.

**Figure 6:**
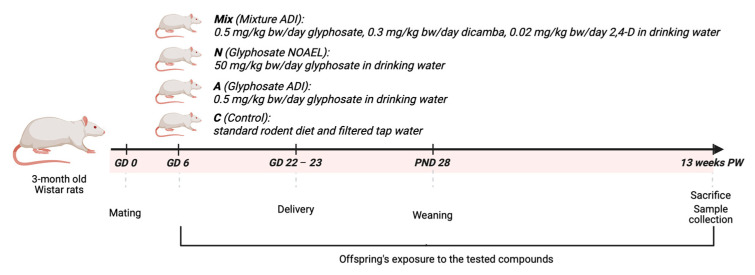
Schematic representation of the experimental design. Offspring were exposed to glyphosate alone and a mixture of glyphosate, 2,4-D and dicamba over the period of gestation, lactation, and 13 weeks post-weaning. GD—gestational day; PND—postnatal day; PW—post-weaning; Glyphosate ADI—glyphosate EU acceptable daily intake; Glyphosate NOAEL—glyphosate EU no-observed-adverse-effect level; Mixture ADI—mixture of glyphosate, 2,4-D, and dicamba, each at their EU acceptable daily intake. Created in BioRender. nechalioti, M. (2026) https://BioRender.com/grlu70e.

The experimental design is summarized in Figure 6. Data on body weight and histological outcomes have been previously described in detail [59,98].

### 4.2. RNA Extraction and Sample Preparation

Total RNA was extracted from 24 rat liver samples (6 from each group; 3 males and 3 females) using the miRNeasy Mini Kit (Qiagen, Hilden, Germany) following the manufacturer’s instructions. The quality of the RNA was assessed via agarose gel electrophoresis, and the quantity was measured with a Qubit 2.0 fluorometer (Thermo Fisher Scientific, Waltham, MA, USA) using the Qubit microRNA Assay Kit (Thermo Fisher Scientific, Waltham, MA, USA). Only samples with high RNA quality and a concentration exceeding 200 ng/μL were included in this study.

For downstream small RNA sequencing analysis, 16 sequencing pools were created, corresponding to two biological replicates per experimental group. Samples were grouped based on sex, treatment, and dosage. For each experimental group, two RNA pools were generated by randomly combining one to two individual RNA samples, which were mixed at equimolar amounts for each pool.

### 4.3. Small RNA Library Construction and Sequencing

Small RNA library construction and sequencing were undertaken under contract with Novogene Co. (Cambridge, UK). Briefly, 3′ and 5′ adapters were ligated to the respective ends of the small RNAs, followed by reverse transcription using a specific primer to synthesize first-strand cDNA. The resulting cDNA was then PCR-amplified to produce double-stranded cDNA libraries. These libraries were subsequently purified and size-selected to enrich fragments with insert sizes ranging from 18 to 40 nucleotides, which correspond to the typical size of mature small RNAs. After library preparation, the quality and concentration of each library were assessed to ensure sufficient yield and complexity for sequencing. The final libraries were sequenced on an Illumina HiSeq 2500 platform (Illumina, San Diego, CA, USA), generating high-throughput small RNA sequencing data for downstream analysis.

### 4.4. Bioinformatics Analysis

Raw sequence reads underwent stringent quality control. Adapter sequences, low-quality reads, and sequences shorter than 18 nucleotides were removed using Trimmomatic [99] with default parameters. The resulting high-quality reads were aligned to the Rattus norvegicus reference genome (Ensembl release 110) using Bowtie2 [100,101]. Known miRNAs were identified using miRBase v20.0 [102] as a reference, with miRDeep2 v2.0.1.3 [103] and sRNAtoolbox 2022 [104] being utilized to predict candidate miRNAs and reconstruct their secondary structures. Reads were further aligned to the RepeatMasker database (http://www.repeatmasker.org, accessed on 26 February 2026) and Rfam [105] to annotate and filter out repetitive elements and other non-coding RNAs, including ribosomal RNA (rRNA), transfer RNA (tRNA), small nucleolar RNA (snoRNA), small nuclear RNA (snRNA), and small cytoplasmic RNA (scRNA). Novel miRNAs were predicted by combining miREvo [106] and miRDeep2 v2.0.1.3 [103] analyses, which assessed the secondary structure, Dicer cleavage sites, and minimum free energy of unannotated small RNA reads. Only candidates supported by consistent read evidence and characteristic hairpin structures were retained. To ensure consistent and non-redundant annotation, the following ranking was applied: known miRNA > rRNA > tRNA > snRNA > snoRNA > repeats > genes > NAT-siRNA > novel miRNA. All novel miRNAs should be considered putative predictions derived from computational analysis and have not been experimentally validated.

### 4.5. Analysis of Differentially Expressed miRNAs and Functional Enrichment of Their Target Genes

Raw miRNA expression counts were normalized to transcripts per million (TPM), calculated by multiplying the read count of each miRNA × 106 and dividing by the total read count of all detected miRNAs. Differential expression analysis was conducted using edgeR v4.0.16 [107] for all sex-based comparisons within each experimental group (Control, ADI, NOAEL, and Mixture). *p*-Values were adjusted using the q-value method [108]. miRNAs with an adjusted *p*-value (q-value) < 0.05 and |log_2_ (foldchange)| > 1 were considered significantly differentially expressed (DE).

The potential biological roles of differentially expressed miRNAs were subsequently investigated through target gene predictions using miRTargetLink 2.0 [54], focusing exclusively on experimentally validated targets. Gene Ontology (GO) enrichment analysis [51,52] and KEGG pathway analysis [53] were performed using ShinyGO 0.82 [109] to further clarify the roles of these gene targets. For both analyses, statistical significance was assessed after correcting for false discovery rate (FDR) to account for multiple comparisons, applying an FDR-adjusted *p*-value threshold of <0.05. Additionally, only the overlapping gene targets of the DE miRNAs were included in both the GO enrichment and KEGG pathway analyses.

## 5. Conclusions

In summary, this study provides a preliminary assessment of sex-dependent divergence in hepatic miRNA expression following perinatal exposure to regulatory relevant (NOAEL and ADI) doses of glyphosate and a mixture of glyphosate, 2,4-D and dicamba across exposure conditions. Baseline differences in miRNA expression were observed between males and females, while the most pronounced divergence was identified in the mixture-exposed group. Patterns of differential miRNA expression suggest that exposure may be associated with distinct sex-dependent regulatory responses, with differences observed in miRNAs linked to pathways involved in cellular regulation, stress responses, and tissue homeostasis. These findings should be interpreted with caution, as they are based on pooled sequencing data and bioinformatic predictions without direct functional validation.

Overall, the present study identifies candidate sex-divergent small RNA signatures associated with herbicide exposure and highlights the potential importance of considering sex a biological variable in developmental and mixture toxicology. Further studies using larger cohorts, individual-level sampling, and functional validation are required to confirm these observations and to better understand their biological and toxicological significance.

## Figures and Tables

**Table 1:** Top differentially expressed miRNAs across comparisons between male and female rats within each treatment group. miRNAs are ranked based on adjusted *p*-values and fold change (log2FC). Positive log2FC values indicate upregulation in males, while negative values indicate downregulation in males relative to females.

Comparison	miRNAs	log2FC	*p*-Value (Adjusted)	Regulation
MC vs. FC	rno-let-7b-3p	−5.64	0.041	Down (Male)
	rno-miR-190b-5p	−3.51	0.022	Down (Male)
	rno-miR-3574	6.24	0.007	Up (Male)
	rno-miR-542-3p	5.6	0.041	Up (Male)
MA vs. FA	novel_156	−2.28	0.007	Down (Male)
	rno-miR-375-3p	−1.69	0.021	Down (Male)
	rno-miR-31a-3p	3.21	0.035	Up (Male)
	rno-let-7c-1-3p	2.76	0.027	Up (Male)
MN vs. FN	rno-miR-672-5p	−6.58	0.007	Down (Male)
	novel_196	−6.36	0.012	Down (Male)
	rno-miR-294	6.22	0.007	Up (Male)
	rno-miR-429	2.74	0.027	Up (Male)
MMix vs. FMix	rno-miR-19b-3p	−8.14	1.16814322597595 × 10^−7^	Down (Male)
	novel_171	−6.52	0.002	Down (Male)
	novel_143	6.85	0.0005	Up (Male)
	rno-miR-224-5p	6.66	0.0008	Up (Male)

## Data Availability

The data presented in the manuscript are available upon reasonable request from the corresponding authors.

## Supplementary Materials

The following supporting information can be downloaded at: https://www.mdpi.com/article/10.3390/ijms27104221/s1.

## Abbreviations

The following abbreviations are used in this manuscript:

ADI	Acceptable Daily Intake
NOAEL	No-Observed-Adverse-Effect Level
EU	European Union
2,4-D	2,4-Dichlorophenoxyacetic Acid
Dicamba	3,6-Dichloro-2-methoxybenzoic Acid
IARC	International Agency for Research on Cancer
GBHs	Glyphosate-Based Herbicides
OECD	Organization for Economic Co-Operation and Development
EPA	Environmental Protection Agency
EFSA	European Food Safety Authority
MRL	Maximum Residue Limit
RLRS	Real-Life Risk Simulation
miRNAs	MicroRNAs
sRNA-seq	Small RNA Sequencing
DE	Differentially Expressed
GO	Gene Ontology
KEGG	Kyoto Encyclopedia of Genes and Genomes
FDR	False Discovery Rate
ECM	Extracellular matrix
SOCS	Cytokine Signaling
rRNA	Ribosomal RNA
tRNA	Transfer RNA
snoRNA	Small Nucleolar RNA
snRNA	Small Nuclear RNA
scRNA	Small Cytoplasmic RNA
TPM	Transcripts Per Million
