# Supplementary figures and images for: Sex-Specific sRNA Signatures in Rat Liver Reveal Divergent Alterations Following Perinatal Exposure to Glyphosate and Its Mixture with 2,4-D and Dicamba
Source: Int J Mol Sci. 2026 May 9;27(10):4221. doi: 10.3390/ijms27104221 (PMC13207187; doi:10.3390/ijms27104221)

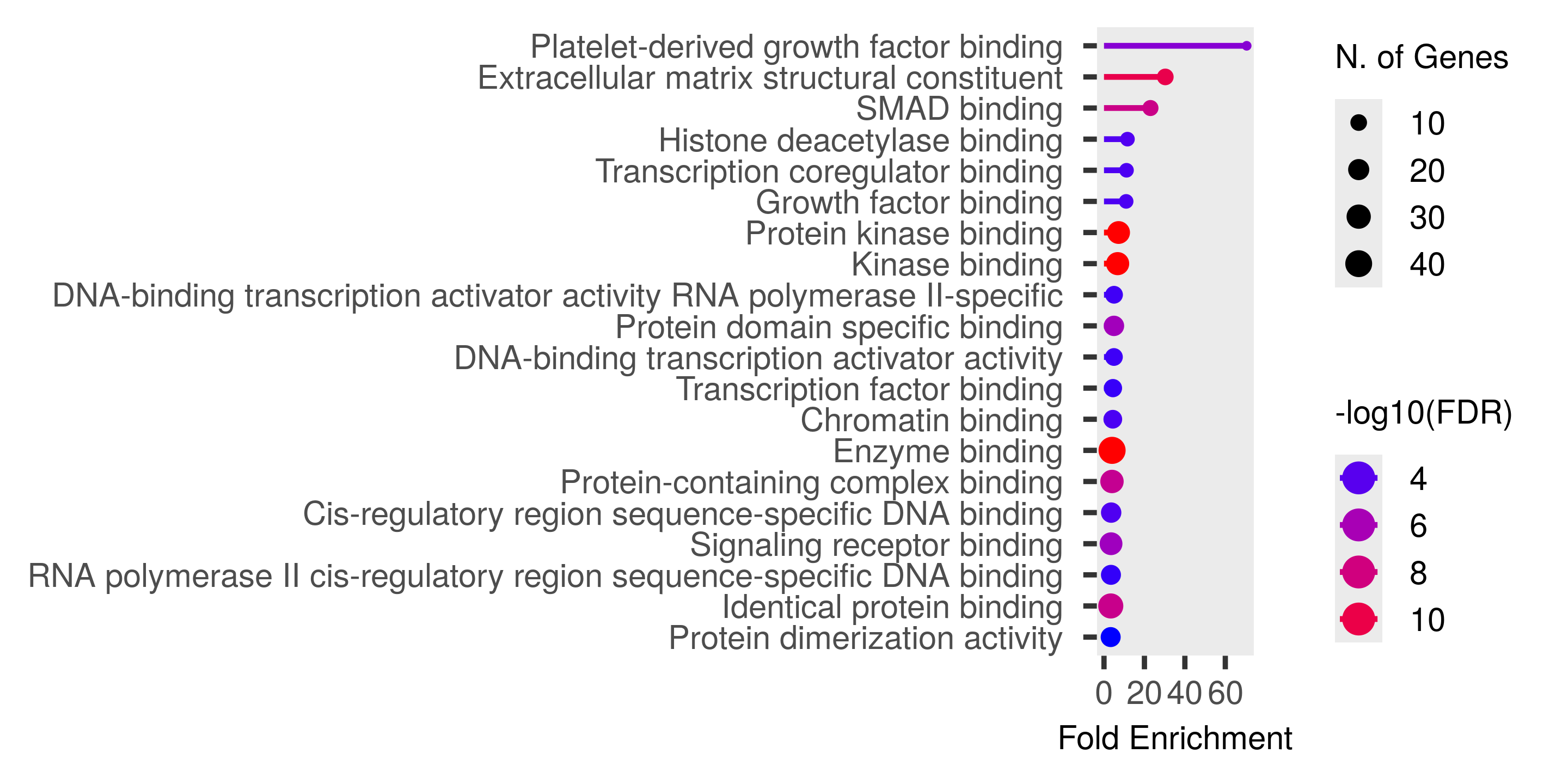

Supplement: Supplementary file 1 [file ijms-27-04221-s001.zip › Supplementary/Figure S6_updated.png]

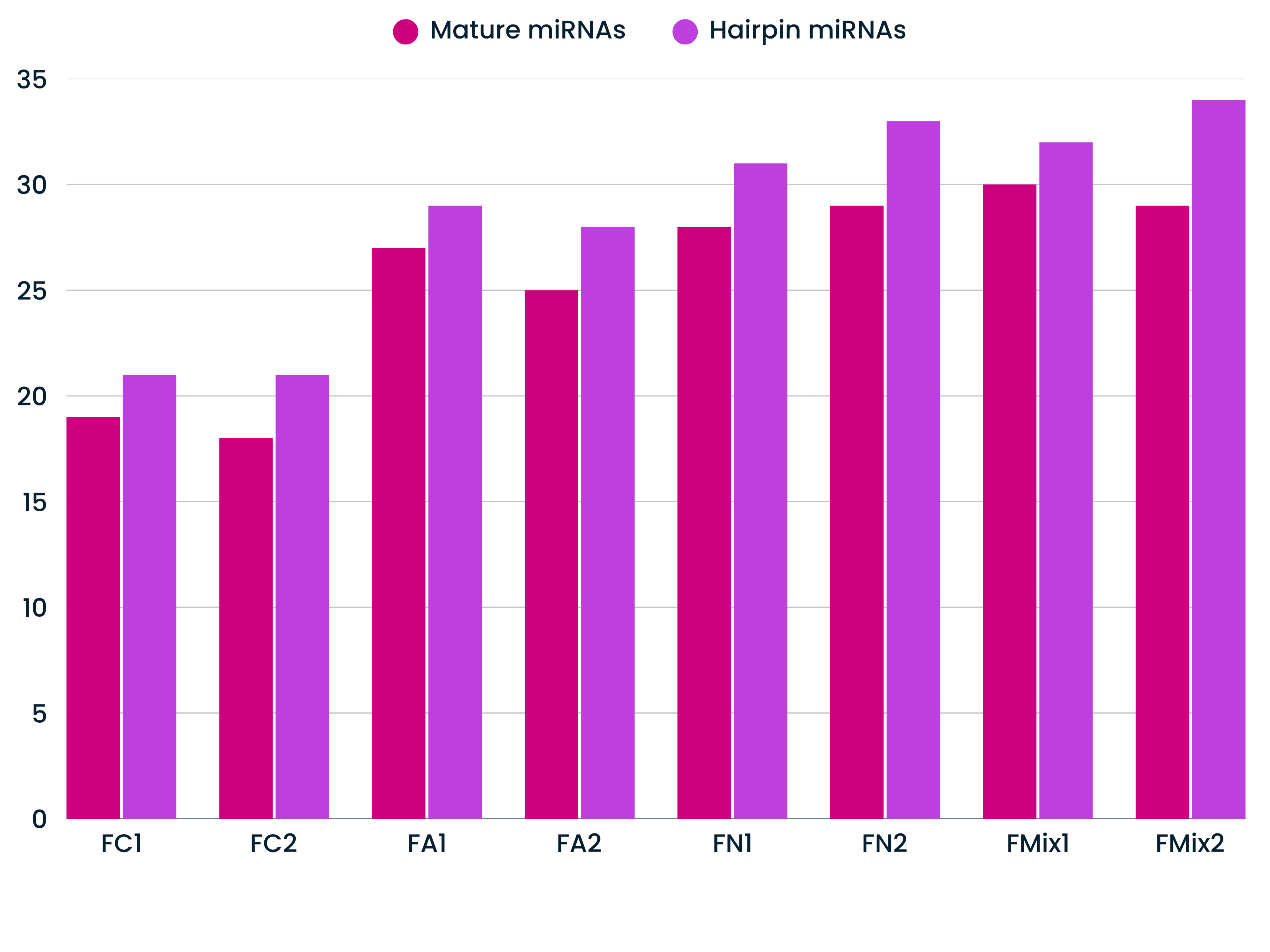

Supplement: Supplementary file 1 [file ijms-27-04221-s001.zip › Supplementary/Figure S3_updated.png]

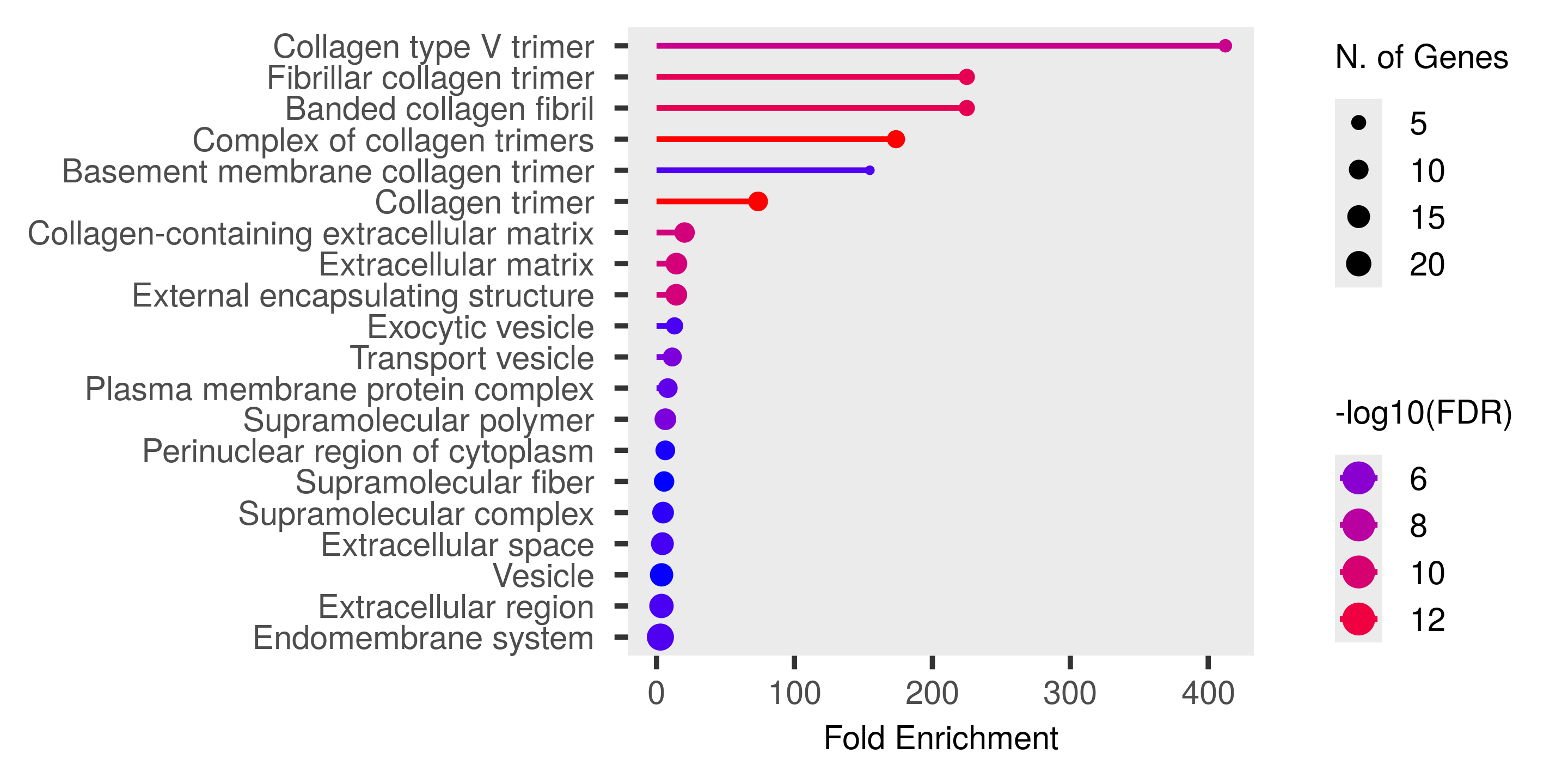

Supplement: Supplementary file 1 [file ijms-27-04221-s001.zip › Supplementary/Figure S9_updated.png]

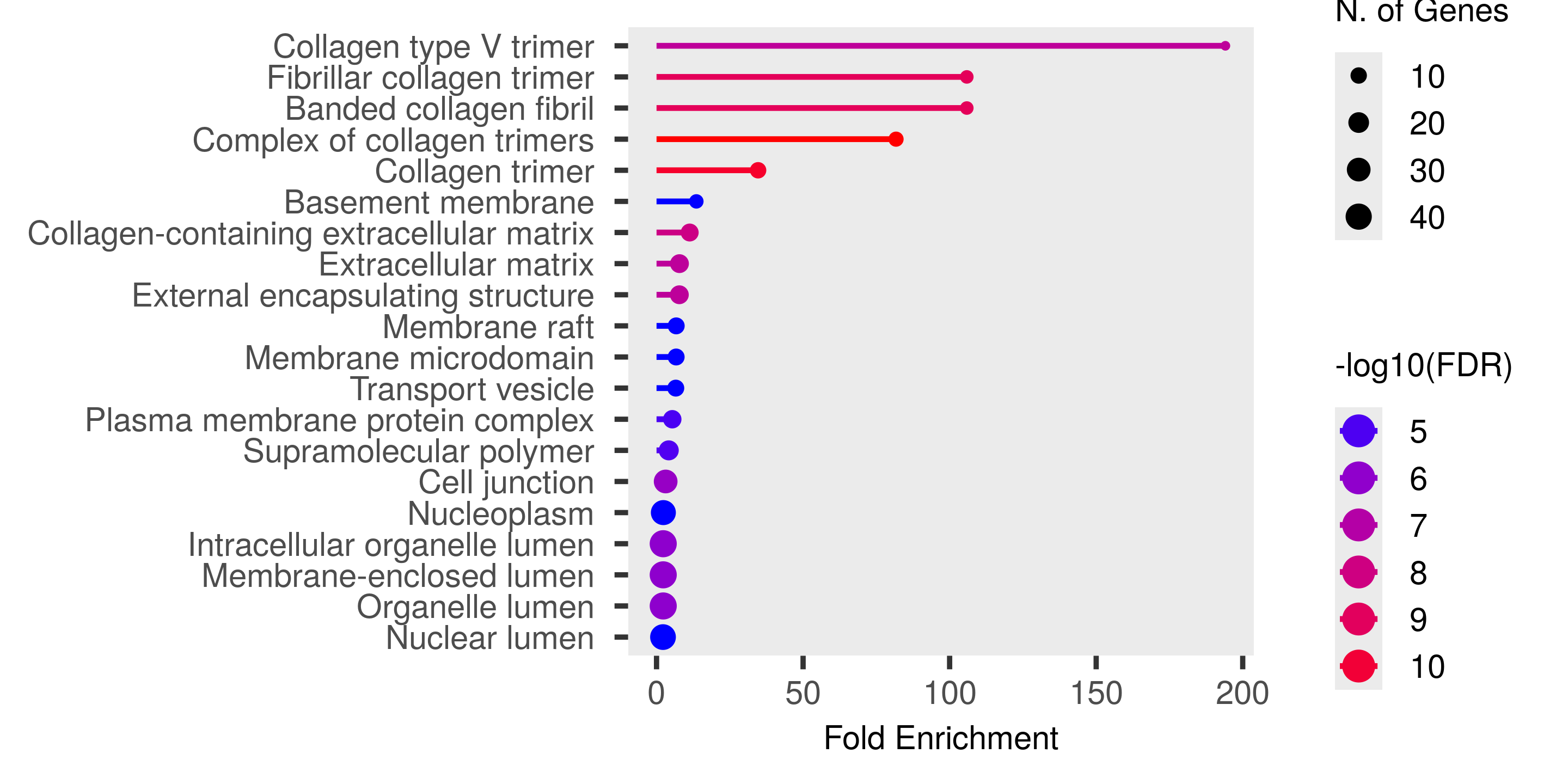

Supplement: Supplementary file 1 [file ijms-27-04221-s001.zip › Supplementary/Figure S5_updated.png]

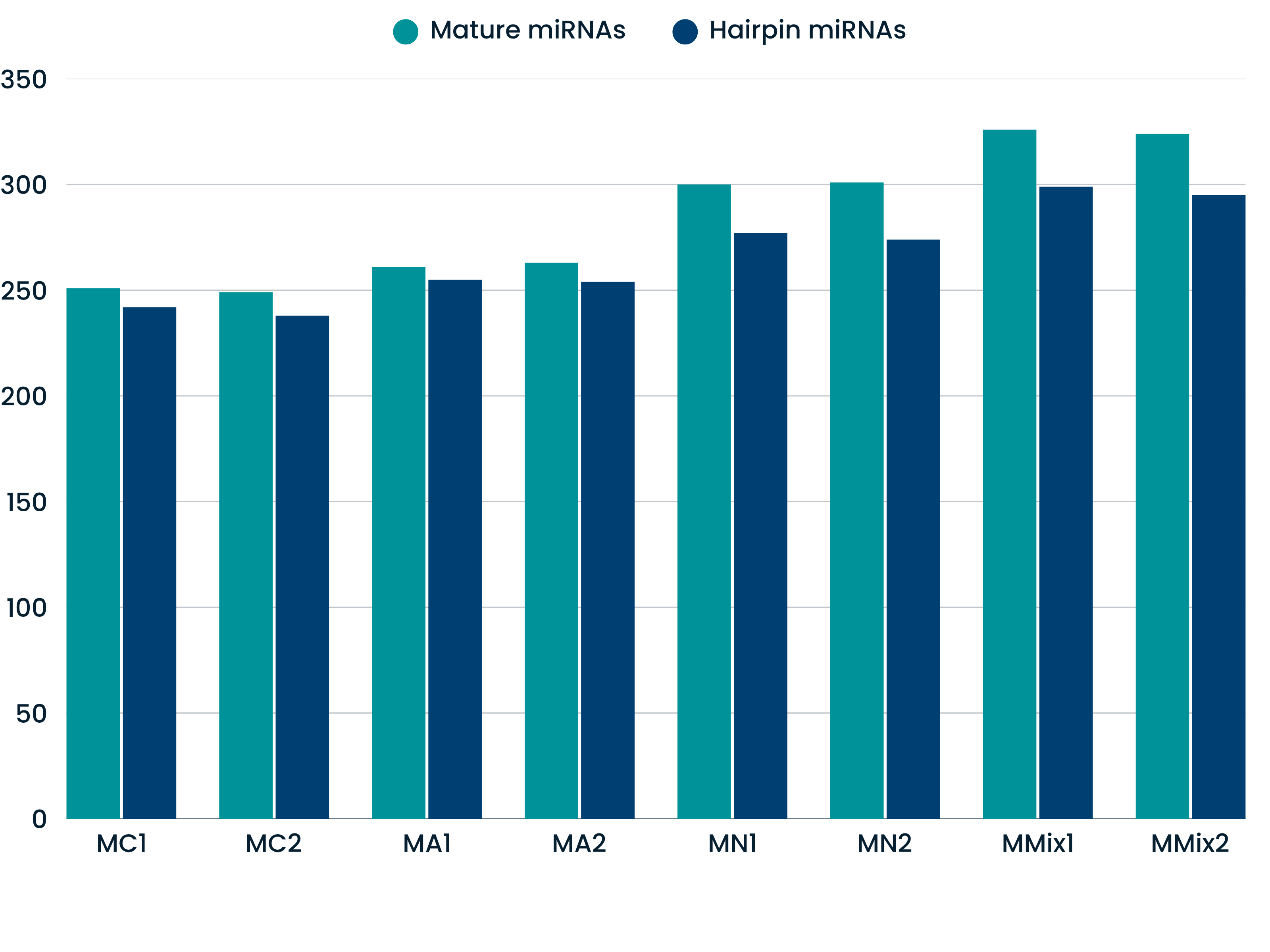

Supplement: Supplementary file 1 [file ijms-27-04221-s001.zip › Supplementary/Figure S2_updated.png]

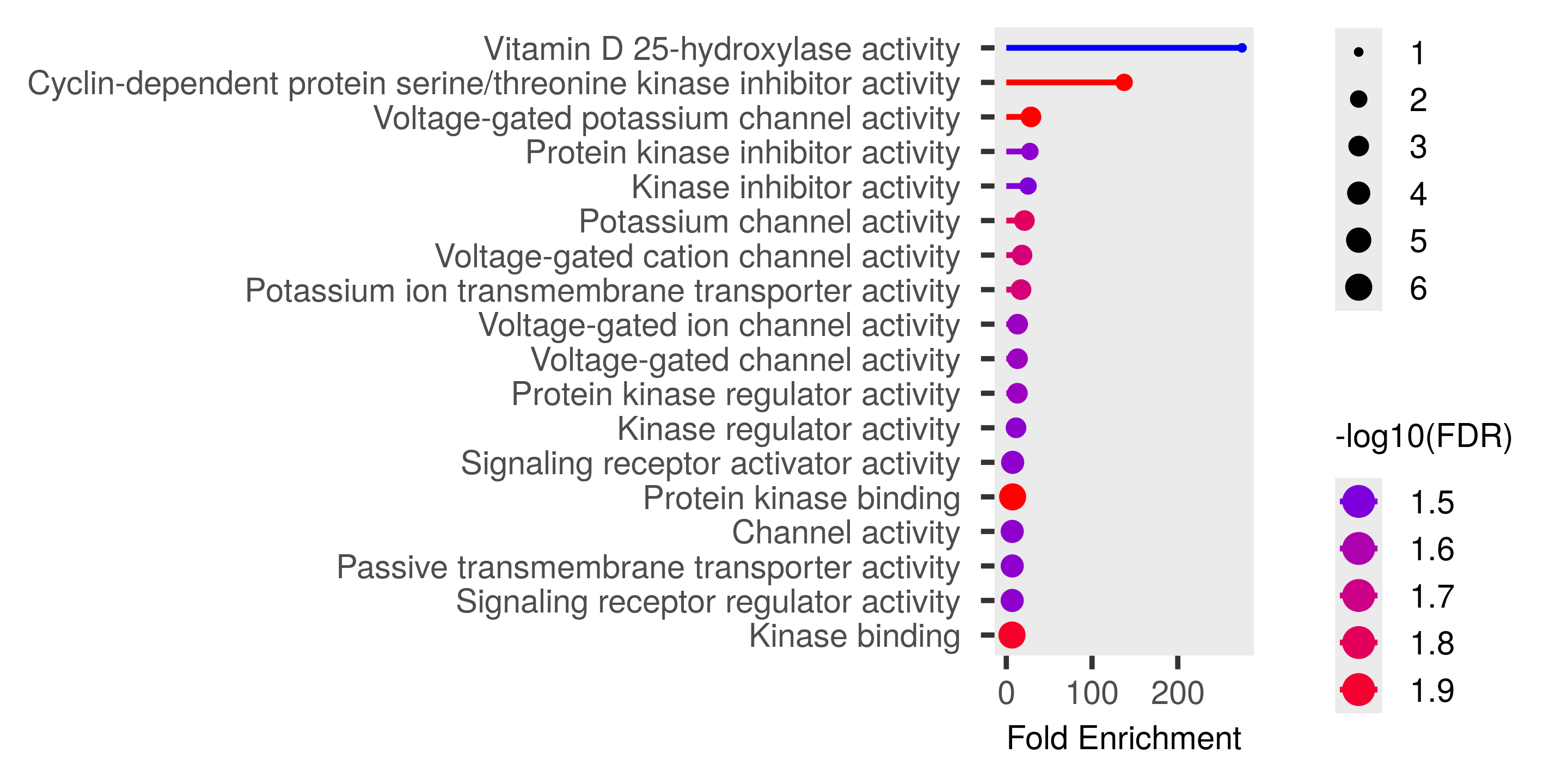

Supplement: Supplementary file 1 [file ijms-27-04221-s001.zip › Supplementary/Figure S7_updated.png]

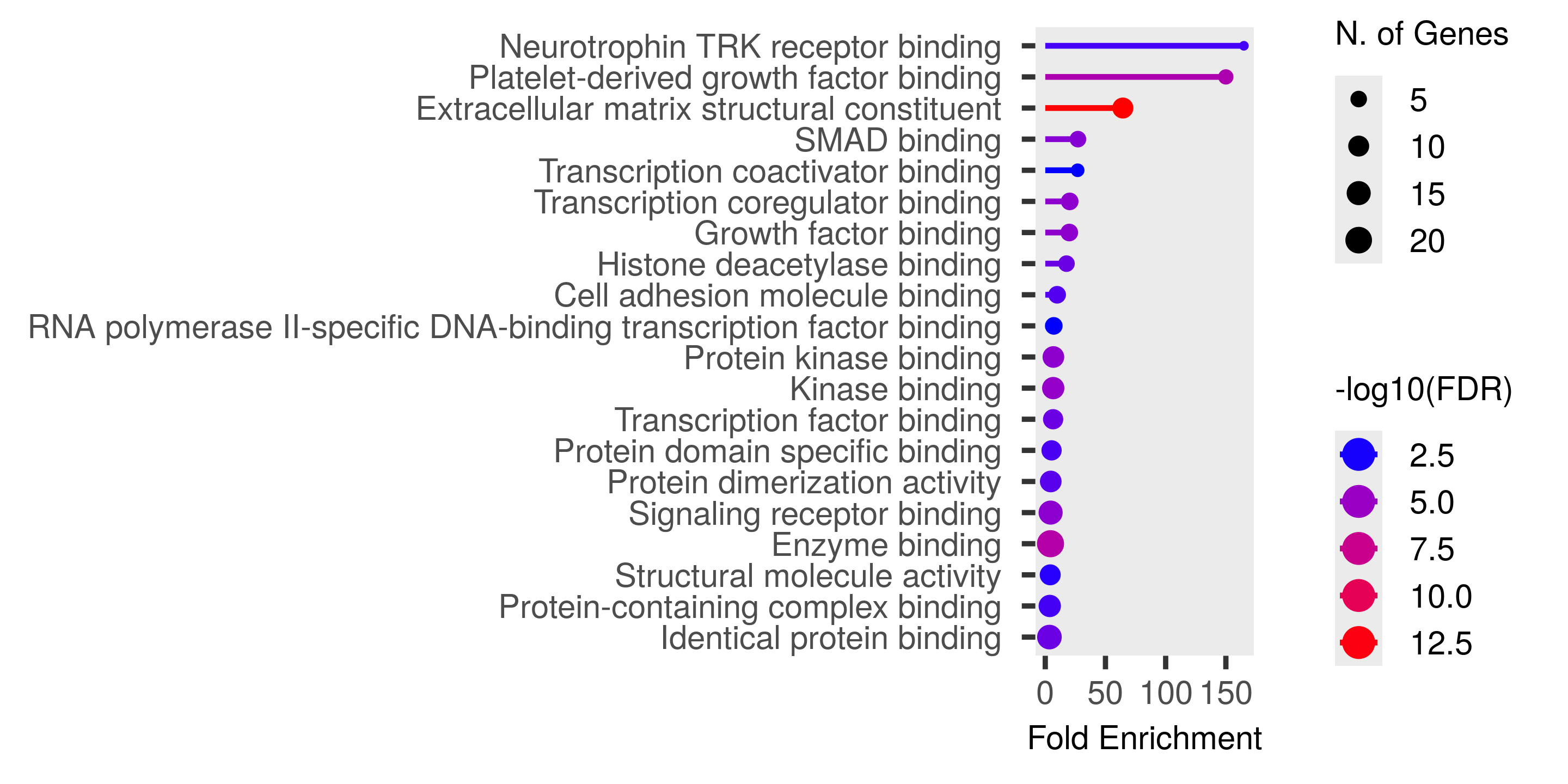

Supplement: Supplementary file 1 [file ijms-27-04221-s001.zip › Supplementary/Figure S10_updated.png]

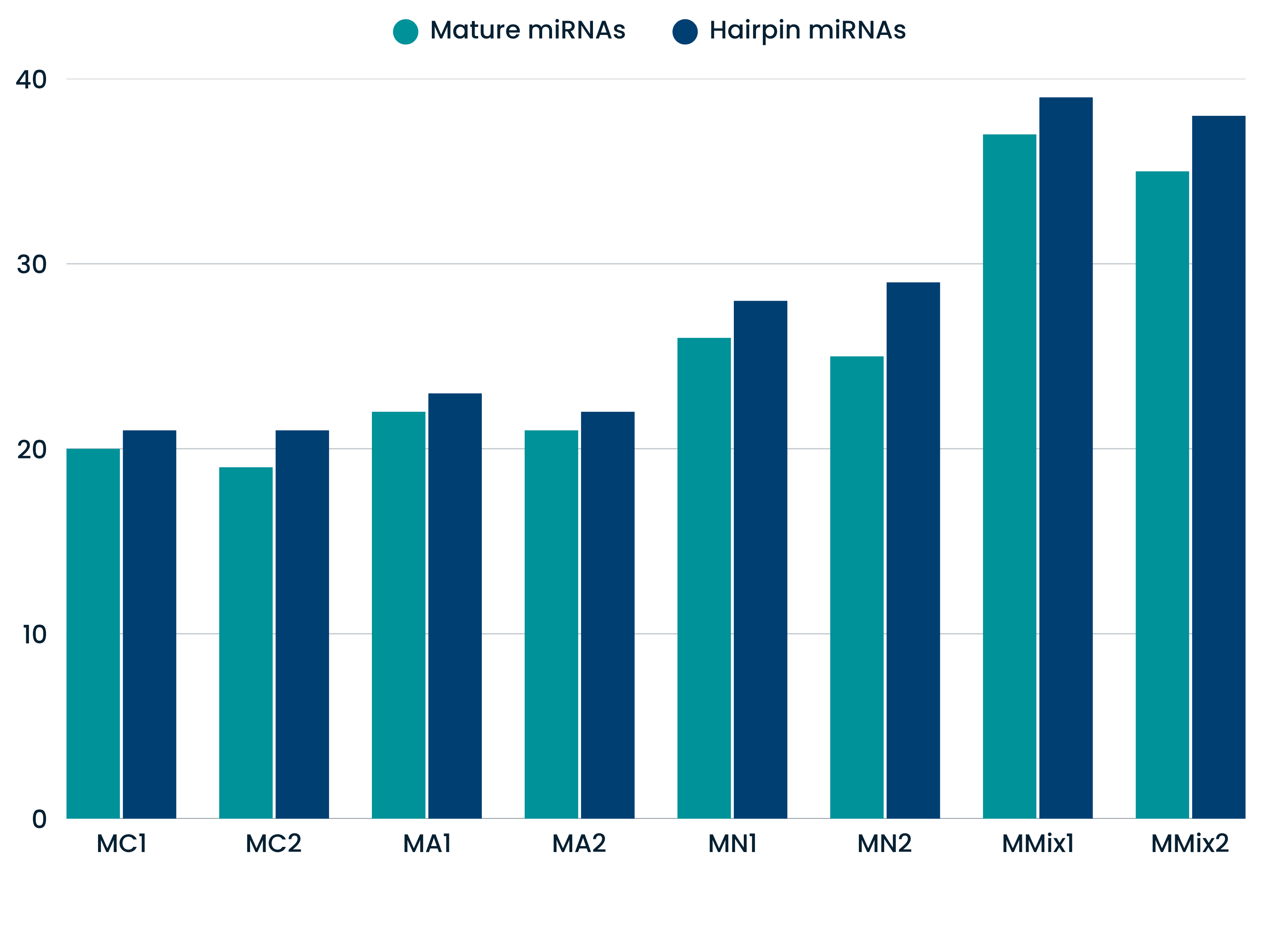

Supplement: Supplementary file 1 [file ijms-27-04221-s001.zip › Supplementary/Figure S4_updated.png]

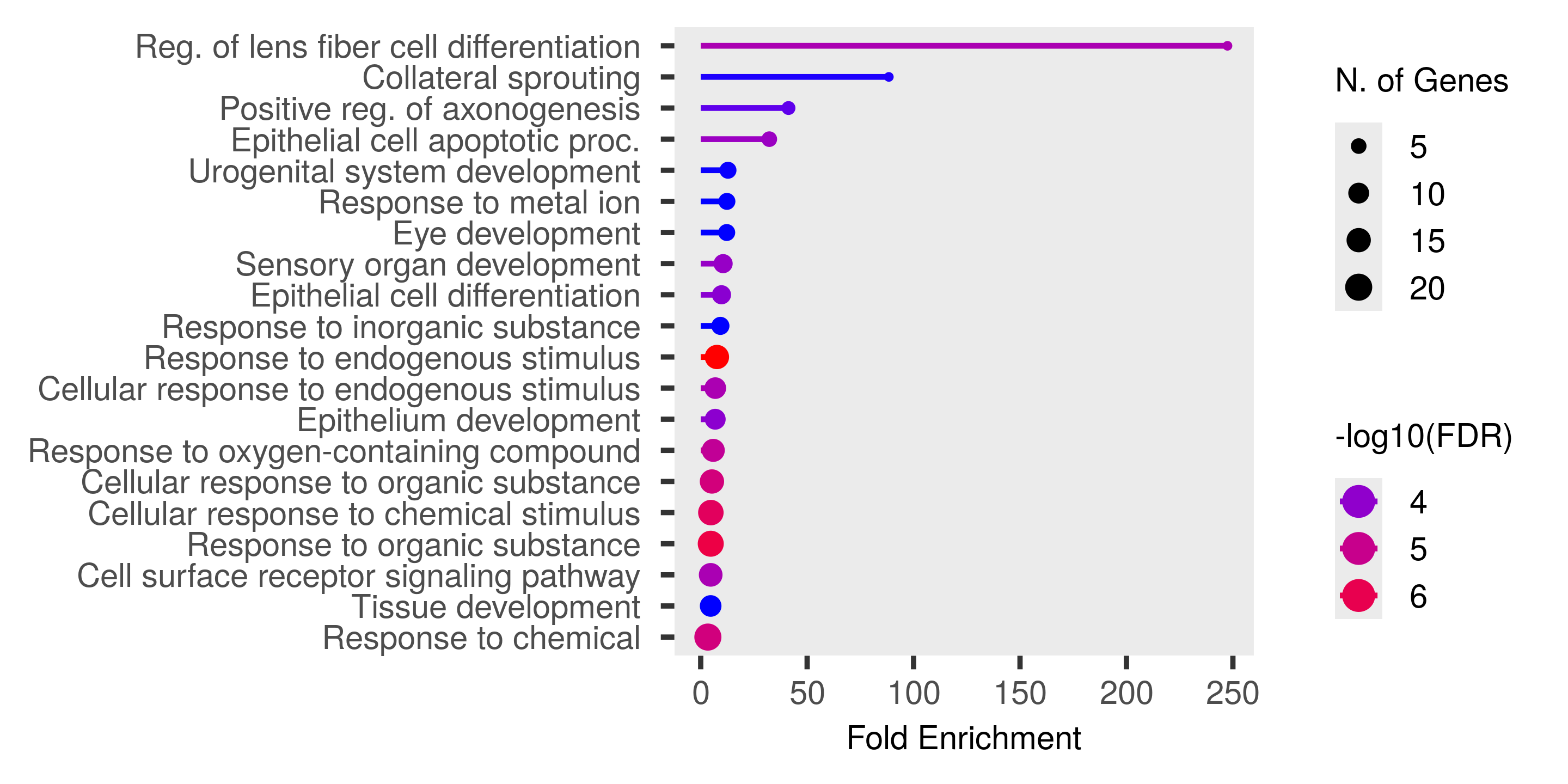

Supplement: Supplementary file 1 [file ijms-27-04221-s001.zip › Supplementary/Figure S8_updated.png]

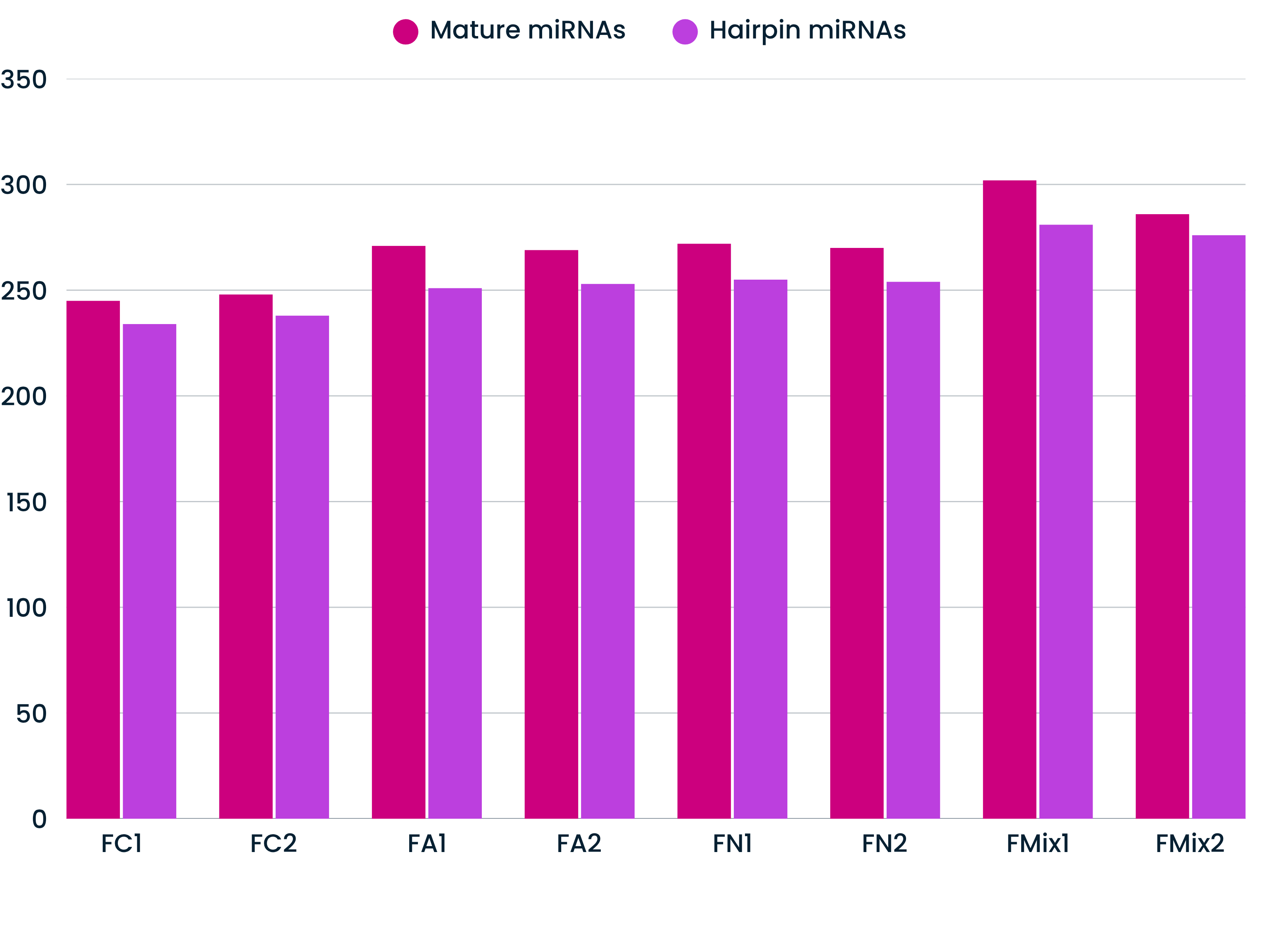

Supplement: Supplementary file 1 [file ijms-27-04221-s001.zip › Supplementary/Figure S1_updated.png]
